# Aldrin: MAK Value Documentation, addendum – Translation of the German version from 2023

**DOI:** 10.34865/mb30900e10_2ad

**Published:** 2025-06-30

**Authors:** Andrea Hartwig, MAK Commission

**Affiliations:** 1 Institute of Applied Biosciences. Department of Food Chemistry and Toxicology. Karlsruhe Institute of Technology (KIT) Adenauerring 20a, Building 50.41 76131 Karlsruhe Germany; 2 Permanent Senate Commission for the Investigation of Health Hazards of Chemical Compounds in the Work Area. Deutsche Forschungsgemeinschaft, Kennedyallee 40, 53175 Bonn, Germany. Further information: Permanent Senate Commission for the Investigation of Health Hazards of Chemical Compounds in the Work Area | DFG

**Keywords:** Aldrin, Insektizid, Pestizid, Toxizität, Bewertung, aldrin, insecticide, pesticide, toxicity, evaluation

## Abstract

Aldrin [309-00-2] is used as an insecticide but is no longer approved in the European Union. The previous MAK value documentation and addendum do not reflect the current data situation of the substance. The MAK Commission decided that a new evaluation is not of high priority. The MAK value and the other classifications are therefore suspended and the substance is listed in the Section II c of the List of MAK and BAT Values for substances no longer evaluated.

**Table:** 

**MAK value**	**see Section II c of the List of MAK and BAT Values**
**Peak limitation**	**–**
	
**Absorption through the skin**	**–**
**Sensitization**	**–**
**Carcinogenicity**	**–**
**Prenatal toxicity**	**–**
**Germ cell mutagenicity**	**–**
	
**BAT value**	**–**
	
Synonyms	aldrite HHDN
Chemical name (IUPAC)	(1S,2S,3S,6R,7R,8R)-1,8,9,10,11,11-hexachlorotetracyclo[6.2.1.1^3,6^.0^2,7^]dodeca-4,9-diene
CAS number	309-00-2
Molecular formula	C_12_H_8_Cl_6_
Molar mass	364.91 g/mol
Melting point	104 °C (NCBI 2023)
Vapour pressure at 20 °C	0.00009 hPa (NCBI 2023)
log K_OW_	6.5 (NCBI 2023)
Solubility	170 mg/l water at 25 °C 0.027 mg/l water at 27 °C (NCBI 2023)

This addendum was prepared because the published evaluation no longer reflects the data currently available for the MAK value and for the designation and classification of the substance.

Aldrin was used as an insecticide to control pests such as termites, locusts and wireworms (UBA 2021 a). Aldrin is converted to dieldrin in plants and animals. In 1966, a MAK value of 0.25 mg/m^3^ I was established for the substance and it was designated with an “H” (for substances which can be absorbed through the skin in toxicologically relevant amounts) (Henschler 1973, available in German only). In 2002, the substance was classified in Peak Limitation Category II with an excursion factor of 8 (Greim 2002, available in German only).

Aldrin is one of the original 12 persistent organic pollutants (POP) that were banned from worldwide production, sale and use under the Stockholm Convention (or “POP Convention”) of 22 May 2001 that came into force on 17 May 2004 (UBA 2021 b). In the European Union, the production, placement on the market and use of aldrin are prohibited under Regulation (EC) No 1107/2009 concerning the placing of plant protection products on the market and Regulation (EU) No 2019/1021 on persistent organic pollutants (European Commission 2022 a; European Parliament and European Council 2009, 2019). Aldrin is also on the list of chemicals in Annex I Part 3 and in Annex II of the PIC (Prior Informed Consent) Regulation (EU) No 649/2012 that have been placed under an export ban (European Commission 2022 b). In the Federal Republic of Germany, approval for the use of aldrin was withdrawn in 1983. In the former German Democratic Republic, use of the substance was permitted until 1979 (BVL 2010).

The previous evaluation does not reflect the currently available data. However, a re-evaluation of the substance is not a priority. Therefore, the MAK value, the peak limitation and the “H” designation have been withdrawn and the substance has been allocated to Section II c of the List of MAK and BAT Values (DFG 2022). This section lists substances for which the previous MAK values, designations and classifications have been withdrawn and which are no longer being reviewed at present.
